# Unimpaired groupitizing in children and adolescents with dyscalculia

**DOI:** 10.1038/s41598-022-09709-5

**Published:** 2022-04-04

**Authors:** Giovanni Anobile, Moreno Marazzi, Stefano Federici, Agnese Napoletti, Lucia Cecconi, Roberto Arrighi

**Affiliations:** 1 Department of Neuroscience, Psychology, Pharmacology and Child Health, University of Florence, Florence, Italy; 2 Clinical Psychology Center “Dedicare”, Foligno, Italy; 3 Developmental Neuropsychology and Speech Therapy Center “Un Mondo di Parole”, Perugia, Italy; 4 Department of Philosophy, Social and Human Sciences and Education, University of Perugia, Perugia, Italy; 5 Department of Developmental Psychology and Socialization (DPSS), University of Padova, Padua, Italy

**Keywords:** Human behaviour, Pattern vision

## Abstract

When asked to estimate the number of items in the visual field, neurotypical adults are more precise and rapid if the items are clustered into subgroups compared to when they are randomly distributed. It has been suggested that this phenomenon, termed “groupitizing”, relies on the recruitment of arithmetical calculation strategies and subitizing. Here the role of arithmetical skills in groupitizing was investigated by measuring the groupitizing effect (or advantage) in a sample of children and adolescents with and without math learning disability (dyscalculia). The results showed that when items were grouped, both groups of participants showed a similar advantage on sensory precision and response time in numerosity estimates. Correlational analyses confirmed a lack of covariation between groupitizing advantage and math scores. Bayesian statistics on sensory precision sustained the frequentist analyses providing decisive evidence in favor of no groups difference on groupitizing advantage magnitude (*LBF* = − 0.44) and no correlation with math scores (*LBF* = − 0.57). The results on response times, although less decisive, were again in favor of the null hypothesis. Overall, the results suggest that the link between groupitizing and mathematical abilities cannot be taken for granted, calling for further investigations on the factors underlying this perceptual phenomenon.

## Introduction

Mathematical abilities vary substantially in the population but the factors that account for such variability are still far from being completely understood. Over the last decades, much evidence suggests that mathematical cognition is highly complex, involving neurocognitive, environmental, and genetic factors^1^. A line of research has focussed on the role of a non-symbolic function that humans, despite being the only species equipped with a symbolic mathematical system, share with several non-human species^2,3^. This function, named “number sense” or “approximate number system”, enables the rapid but approximate perception of numerical quantities: numerosity^4,5^. Typically, an experiment designed to measure this function requires participants to name the perceived number of objects populating the visual field or to select the most numerous ensemble amongst different options. To avoid counting and, on the contrary, ensure a rapid and instantaneous perception, visual objects are usually presented for just few milliseconds. In contrast to serial counting, approximate numerosity estimation is fast but prone to errors. Precision, accuracy, and response time are usually considered gold standards for measuring estimation performance. Precision is usually indexed as the standard deviation of the responses normalized by the physical or the perceived number of objects (Weber's fraction or coefficient of variation, respectively), a measure of numerosity sensitivity. Accuracy refers to the magnitude of the offset between the provided responses and the target numerosity, an index of bias. Finally, response speed reflects the time needed to process incoming sensory information and take related decisions. As with formal math abilities, numerosity perception proficiency largely differs between individuals^6^ and it has been shown that individual differences in numerosity sensitivity (Weber fractions) measured in 14-year-olds correlate with participants’ maths achievement scores measured in the previous years^7^. Several studies replicated the finding of a correlation between precision in numerosity tasks and proficiency in math learning^8–10^ and added that individuals with developmental dyscalculia, a learning disorder limiting math acquisition, also show a deficit for numerosity sensitivity^1,11–13^.

A widely debated question regards the nature of the link between numerosity perception and cognitive mathematical abilities. A recent hypothesis points to the phenomenon termed “groupitizing”. Groupitizing has been originally defined as “the ability to capitalize on grouping cues to facilitate enumeration processes”^14^. Basically, when an array of visual items is spatially clustered into a few (usually < = 4) sub-groups, with each group containing a few items (usually < = 4), the counting speed increases compared to enumeration of randomly scattered items^14,15^, suggesting the groupitizing “advantage” might reflect the use of basic mathematical strategies. For example, when a group of nine objects is divided into three groups of three items and observers are required to report the total numerosity, a viable strategy—provided they have sufficient mathematical skills—is to perform a simple addition like 3 + 3 + 3 or a multiplication 3 × 3. In line with this idea, the groupitizing advantage (the difference in performance between grouped and unstructured stimuli) in children increases with school grade and math competence, with no groupitizing advantage found in pre-schoolers^14^. According to the existent literature, the groupitizing phenomenon is consistent, robust, and generalized. For example, it has been recently shown that counting speed increases when items were grouped by color, and university students with higher math skills performed significantly faster compared to relatively lower math skilled participants^16^. Groupitizing might not only affect counting speed but also the sensitivity for approximate estimates as shown by studies reporting that spatial and color grouping both boosted estimation precision (Coefficient of variation) and response time^17–20^ in approximate numerosity estimation tasks.

Even if the presence of grouped items in the scene yields groupitizing effects and these, in turn, are induced by mathematical strategies, it cannot be excluded that participants implicitly operate clustering and summation/multiplication strategies for unstructured arrays as well. These automatic math strategies, applied to achieve the best possible estimate of stimuli numerosity, might play a key role for the often-observed correlation between numerosity acuity and mathematical proficiency. A direct prediction of this hypothesis is that individuals with higher mathematical ability should benefit more from stimulus clustering when performing an approximate estimation task. To date, there are only two reports in the literature supporting this idea and both involve neurotypical university students. The first study showed a positive correlation between the groupitizing advantage on numerosity precision (coefficient of variation) and arithmetic abilities^19^, suggesting an interplay between the two dimensions. Unfortunately, this study suffers from some limitations such as a reduced sample size (N12) and a mono-dimensional assessment of mathematical skills (one task measuring simple mental calculations) which require the results to be interpreted with caution. A second study investigated this issue on a larger sample of university students^20^. Here, the participants were divided into three groups according to their level of mathematical ability as defined by their college entrance scores. The results showed that the magnitude of the advantage induced by groupitizing on numerosity estimation precision was similar across the three groups. However, the groupitizing advantage on response time was larger in the higher math ability group, compared to the others. Although in line with the idea that groupitizing would reflect math strategies, this study also suffers from some limitations. For example, the categorical inclusion of participants into sub-groups according to college entrance scores might not have been sensitive enough to deeply describe arithmetic abilities, so that the impact of math on the groupitizing effect might have been underestimated. Moreover, the null effect on sensory precision casts some doubts on the link between groupitizing and arithmetical abilities. Taken together these studies, although encouraging, leave the issue largely open and needing further investigation. To this aim, with a numerosity estimation task meant to tap into the “number sense”, we psychophysically measured the groupitizing effect on accuracy, precision, and response time. To promote a wide inter-individual variability in math skills, we recruited and tested a group of children and adolescents with and without a diagnosis of developmental dyscalculia. The groupitizing effects were then compared across groups and correlated with an aggregate index of math abilities measured through a comprehensive neuropsychological battery designed for the diagnosis of dyscalculia (9 different sub tests). If the groupitizing advantage arises from the use of math strategies, we expect lower advantage in individuals with dyscalculia.

## Results

**Figure 1:**
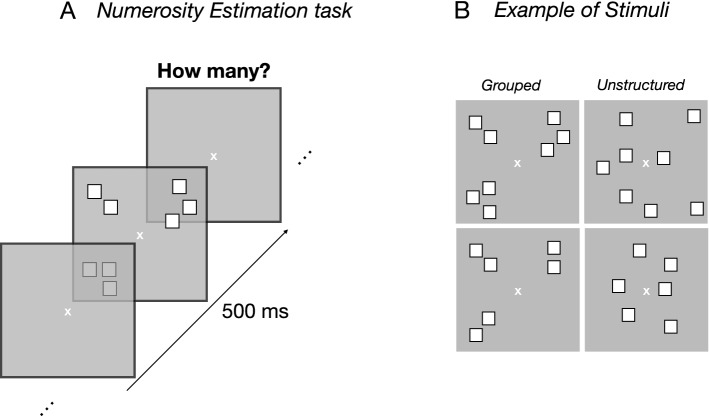
Stimuli and procedure. (**A**) Example of the time course of the experiment. Participants were asked to verbally report the number of the perceived numerosity. (**B**) Examples of stimuli arrangement for numerosity 8 and 6. Stimuli are not depicted to scale.

To investigate the role of math abilities in the groupitizing effect we asked participants with and without dyscalculia to estimate the number of quickly presented visual arrays. Items were either randomly scattered in space (unstructured) or spatially grouped into a few groups, each containing a few items (Fig. 1). Data were analyses by frequentist and Bayesian statistics (Log10 Bayes Factors, *LBF*). By convention, *LBF* provide weak (0–0.5), substantial (0.5–1), strong (1–2) or decisive (> 2) evidence in favour of the alternative hypothesis (H1) with the same critical, but negative, values providing support to the null hypothesis (H0).

### Neuropsychological tests

Table 1 shows average scores obtained by the two groups in the neuropsychological tests. As expected, the group with dyscalculia performed poorly in the math tasks (*p* < 0.001, *LBF* > 3). As mentioned in the methods section, most participants with dyscalculia also met criteria for dyslexia, indicated here by overall slower word reading speed, compared to controls (*p* = 0.009, *LBF* = 0.7). The group with dyscalculia also showed lower non-verbal reasoning abilities (see “Methods” for details, *p* < 0.001, *LBF* = 2.7).

**Table 1:** Descriptive statistics of scores obtained in the neuropsychological tests.

Measure	Dyscalculia	Controls	*t* test
Math aggregate index (z-score)	*M* = − 1.55 *SD* = 0.5	*M* = 0.16 *SD* = 0.58	*p* < 0.001 *LBF* > 3
Word reading accuracy (z-score)	*M* = 0.62 *SD* = 2.33	*M* = 0.37 *SD* = 0.58	*p* = 0.65 *LBF* = − 45
Word reading speed (z-score)	*M* = − 0.93 *SD* = 1.2	*M* = − 0.03 *SD* = 0.59	*p* = 0.009 *LBF* = 0.7
Non-verbal reasoning	*M* = 94.9 *SD* = 9.7	*M* = 110 *SD* = 9.68	*p* < 0.001 *LBF* = 2.7

Independent samples *t* tests with associated two-tailed p-values and Log10 Bayes Factors (*LBF*).

### Groupitizing and perceived numerosity

**Figure 2:**
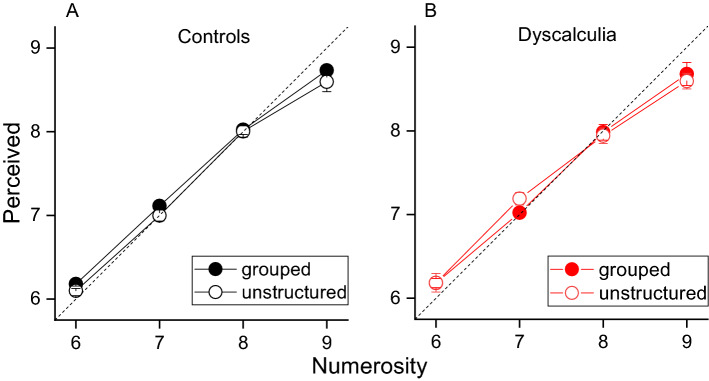
Perceived numerosity. Average perceived numerosity as a function of physical numerosity for the two experimental conditions (empty circles: randomly spaced stimuli, filled circles: grouped stimuli) divided by groups [(**A)** controls, (**B**) dyscalculia]. Error bars are ± 1 SEM.

We first asked whether grouped items lead to accuracy biases. Figure 2 shows average perceived numerosity as a function of physical numerosity for unstructured (filled circles) and spatially grouped stimuli (open circles) separately for controls (A) and dyscalculic participants (B). From inspection it is evident that both groups accurately estimated the number of items, with no biases induced by grouping. A RM ANOVA on responses, with numerosity and spatial configuration as RM factors and groups as between subject factor, confirmed a null effect for configuration (*F*(1,108) = 1.257, *p* = 0.27, *η*^2^ = 0.03, *LBF* = − 1) and for the interaction of configuration by group (*F*(1,108) = 2.16, *p* = 0.15, *η*^2^ = 0.05, *LBF* = − 1.44). Also the factor group was not statistically significant, suggesting similar accuracy between groups (*F*(1,36) = 0.003, *p* = 0.95, *η*^2^ < 0.001, *LBF* = − 1). Overall, these analyses revealed that spatial grouping had no effects on the accuracy of numerosity estimation, neither for controls nor dyscalculics.

### Groupitizing and estimation precision

**Figure 3:**
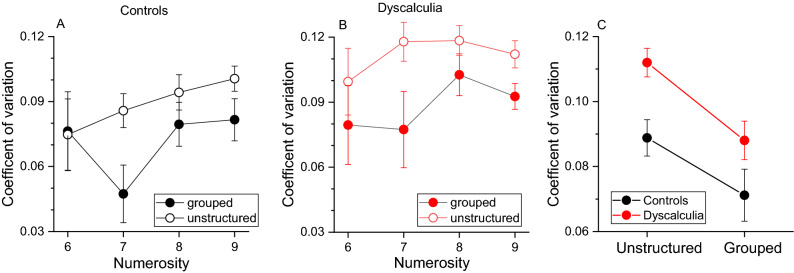
Groupitizing effect on estimation precision. (**A**,**B**) Average coefficient of variation as a function of numerosity for the two experimental conditions (empty circles: randomly spaced stimuli, filled circles: grouped stimuli). (**C**) Coefficients of variation averaged across numerosity levels and participants. Error bars show ± 1 SEM.

Figure 3A,B shows between subjects’ average estimation precision (*CV*, coefficient of variation) separately for each numerosity and stimuli configuration in the control (A) and dyscalculia (B) group. From visual inspection it is evident that both groups show lower *CV* values for grouped compared to unstructured stimuli, indicating that groupitizing leads to higher estimation precision. Figure 3C shows *CVs* averaged across numerosity levels and participants. In the control group the average *CV* for unstructured stimuli was 0.088 decreasing to 0.071 for grouped stimuli. In the group of participants with dyscalculia, the average *CV* for unstructured stimuli was 0.11 and decreased to 0.09 for the grouped condition. A RM ANOVA confirmed a main effect of stimuli configuration (*F*(1,108) = 21.473, *p* < 0.001, *η*^2^ = 0.37, *LBF* = 3.63), indicating higher precision for grouped stimuli across groups. The configuration by group interaction was not statistically significant (*F*(1,36) = 0.49, *p* = 0.5, *η*^2^ = 0.008, *LBF* = − 0.63) as well as the factor group (*F*(1,36) = 2.52, *p* = 0.121, *η*^2^ = 0.066, *LBF* = − 0.4), indicating a similar performance.

Given the previous literature indicating poorer estimation precision in participants with dyscalculia (see “Introduction”), we looked at possible groups differences on *CVs* for unstructured and grouped stimuli separately (RM ANOVAs). The analyses revealed a marginally significant difference only for unstructured stimuli (unstructured: *F*(1,36) = 4.2, *p* = 0.04, *LBF* = 0.06; grouped: *F*(1,36) = 1.26, *p* = 0.27, *η*^2^ = 0.03, *LBF* = − 0.4).

**Figure 4:**
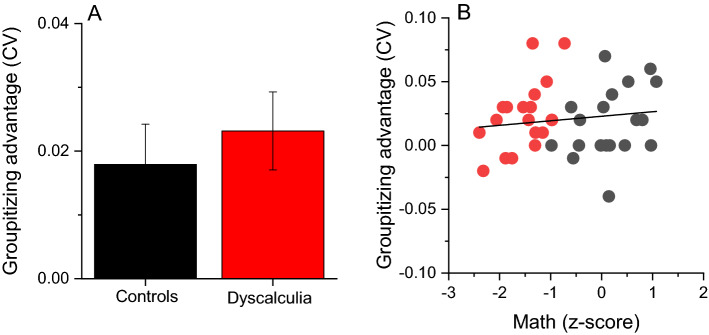
Groupitizing advantage on sensory precision. (**A**) Average standardized difference between precision (*CV*, Eq. 1) for grouped and ungrouped stimuli in the sample with dyscalculia and controls. Error bars show ± 1 SEM. (**B**) Linear correlation between groupitizing advantage on sensory precision and math scores.

To directly compare the magnitude of the groupitizing advantage on estimation precision between the two groups, we computed an “advantage index” for each participant, as the difference between *CVs* in the unstructured and grouped condition (Eq. 2), with positive values indicating lower *CVs* for grouped stimuli. Figure 4A clearly confirmed that, on average, the magnitude of groupitizing advantage was robust but similar across the groups (*t*(36) = 0.59, *p* = 0.55, *d* = 0.19, *LBF* = − 0.44).

Finally, since clinical inclusion criteria depends on arbitrary categorical cut-offs, we also looked at inter-individual differences in the overall mathematical abilities, leaving aside the diagnostic labelling. Figure 4B shows that the groupitizing effect on sensory precision explained a small and not significant portion of math abilities variance (Fig. 4B, r = 0.13, *p* = 0.43, *LBF* = − 0.57). Overall, these analyses revealed that the dyscalculia group, similarly to the control group, do take advantage of groupitizing to improve sensory precision.

### Groupitizing and response time

Previous studies showed that groupitizing could also speed up response time^17,20^. If the advantage on response time (*RT*, hereafter) for grouped stimuli is induced by the optimal use of arithmetic strategies, we expect a smaller advantage in subjects with lower math abilities.

**Figure 5:**
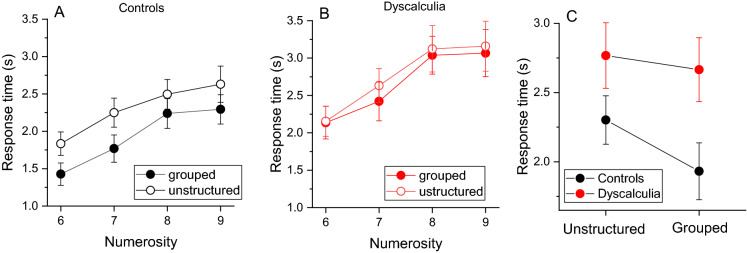
Groupitizing effect on response time. (**A**,**B**) Average response time as a function of numerosity for the two experimental conditions (empty circles: randomly spaced stimuli, filled circles: grouped stimuli). (**C**) Response time averaged across numerosity levels and participants. Error bars show ± 1 SEM.

Figure 5A,B shows between subjects’ average response time (*RT*) separately for each numerosity and stimuli configuration in the control and dyscalculia group. In the control group (A) *RTs* were lower for grouped stimuli, compared to unstructured arrays, confirming that groupitizing accelerated response speed. This effect was much less evident in the group with dyscalculia (B). As shown in Fig. 5C, while in the control group the average *RT* time in the unstructured condition was higher compared to that for unstructured arrays (2.3 s vs 1.9 s respectively), in the group with dyscalculia the *RTs* were similar across stimuli configurations (2.7 s and 2.6 s for unstructured and grouped stimuli), suggesting a weak improvement.

A RM ANOVA on response time confirmed a main effect of the factor configuration (*F*(1,108) = 6.7, *p* = 0.014, *η*^2^ = 0.15, *LBF* = 2.3), indicating an overall lower response time in the grouped condition across the two groups. Importantly, the configuration by group interaction was not statistically significant (*F*(1,36) = 2.16, *p* = 0.15, η^2^ = 0.048) suggesting a similar pattern of results in the two groups across tasks. The *LBF* associated with the interaction was 0.4, indicating anecdotal evidence. Finally, the factor group was marginally significant, suggesting slightly slower *RT* time in the dyscalculia group (*F*(1,36) = 4.25, *p* = 0.046, *η*^2^ = 0.10, *LBF* = 0.3).

**Figure 6:**
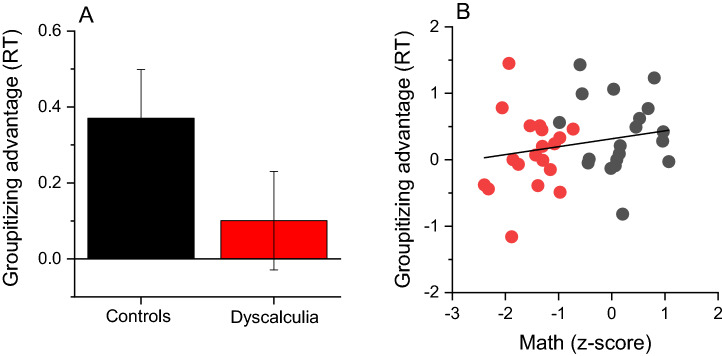
Groupitizing advantage on response time. (**A**) Average standardized difference between response time (*RT*, Eq. 1) for grouped and ungrouped stimuli in the sample with dyscalculia and controls. Error bars show ± 1 SEM. (**B**) Linear correlation between groupitizing advantage on response time and math scores.

As for the analysis on estimation precision, to compare the magnitude of the groupitizing advantage on *RT* between the groups, we computed an “advantage index” (Eq. 1). Again, positive values indicate lower *RTs* in the grouped condition. Figure 6A shows that the magnitude of groupitizing advantage on *RT* was similar across groups (*t*(36) = 1.48, p = 0.147, *d* = 0.48, *LBF* = − 0.13) and not correlated with math abilities (Fig. 6B, r = 0.2, *p* = 0.2, *LBF* = − 0.36).

Finally, as the composite math index also included performance on tasks not directly related to calculation (e.g. counting) likely to be minimally involved in arithmetic-driven groupitizing strategies, we looked at the correlations between groupitizing advantage indexes (separately for *CV* and *RT*) and scores on the mathematical tasks requiring calculation (mental multiplication, mental calculation, fast mental calculation, and approximate mental calculation, see “Methods” for details). The results confirmed the overall pattern on results with none of the arithmetic tasks correlating with groupitizing advantage indexes (Table 2).

**Table 2:** Correlation between groupitizing advantage and arithmetic scores.

	Groupitizing advantage on *CV*	Groupitizing advantage on *RT*
Mental multiplication	*r* = 0.054 *p* = 0.745 *LBF* = − 0.67	*r* = 0.228 *p* = 0.169 *LBF* = − 0.3
Mental calculation	*r* = 0.089 *p* = 0.595 *LBF* = − 0.63	*r* = 0.123 *p* = 0.463 *LBF* = − 0.58
Fast mental calculation	*r* = 0.076 *p* = 0.649 *LBF* = − 0.65	*r* = 0.082 *p* = 0.626 *LBF* = − 0.65
Approximate mental calculation	*r* = 0.058 *p* = 0.729 *LBF* = − 0.67	*r* = 0.049 *p* = 0.772 *LBF* = − 0.67

Pearson correlations with associated two-tailed p-values and Log10 Bayes Factors (*LBF*).

## Discussion

The mechanisms underlying groupitizing are still a matter of debate. The literature suggests that it might reflect the combined use of arithmetical strategies and subitizing (from which it takes part of its name). In the current study, we focused on the role of mathematical abilities. To this aim, a group of children and adolescents with and without a diagnosis of developmental dyscalculia was tested with a psychophysical numerosity estimation task (“how many?”) as well as a battery of mathematical tests. The results replicated previous findings about a robust improvement in precision and response speed for spatially grouped relative to spatially unstructured items (i.e. groupitizing effect). The magnitude of the groupitizing advantage (difference between performance for grouped and unstructured stimuli, see “Methods”) was than compared across the two groups and correlated with an aggregate index of mathematical ability.

Despite the sharp difference in mathematical scores between the two groups (LBF > 3), the groupitizing effect on estimation precision (coefficient of variation) was almost identical between dyscalculic and controls participants (LBF = − 0.44) and clearly not correlating with math scores (LBF = − 0.57). These results provide decisive evidence for an independence between mathematical ability and groupitizing, on estimation precision. The results replicate (and extend to a younger and clinical sample) a previous report on a cohort of typically developing university students^20^. In the latter study, the authors found that high, medium, and low math university students similarly do take advantage from grouped visual items, to improve numerosity estimation precision.

The results obtained here on response time, while again indicating no links with math, were less decisive. The LBF associated with groups difference on the magnitude of groupitizing advantage as well as LBF describing the correlation strength with math scores were both negative (− 0.13, − 0.36 respectively). Negative values indicates that H0 (no difference, no correlation) was more likely that H1. The absolute values of these LBF, however, do not support decisive evidence for H0 (LBF < − 0.5) not allowing definitive conclusions.

Partially at odds with current results, Pan and colleagues^20^ found that only high math university students showed a groupitizing advantage on response time. The study indicates that the groupitizing advantage on response time, compared to estimation precision, was more modulated by math abilities. However, it should be noted that even in this latter study, the groupitizing advantage on response times between medium and low math students was similar, indicating a rather coarse discriminative power. Supporting the idea of a link between groupitizing and mathematical operations, recent imaging data on neurotypical adults, showed that estimates for grouped stimuli elicit selective responses in the left hemisphere, in particular in areas including the lateral and inferior part of the IPS that has been previously reported to be involved in calculation^21^.

With the current data we can only speculate on the mechanism underlying the grouptizing phenomenon and on the reasons why it might not be related to mathematical abilities. A first possibility concerns the relatively advanced developmental stage of our participants. Since groupitizing requires the ability to perform very simple operations with operands within 4, even individuals with low mathematical abilities (especially when tested at a relatively advanced age) might have already developed compensatory strategies to accomplish these tasks, probably at the expense of response speed. The not so clear-cut null results found here on response times, together with the results obtained by Pan and colleagues^20^ on adults are in line with this idea. Response times might, therefore, be more predictive parameter for young-adults’ samples as they (might) better represent the suboptimal use of mathematical strategies.

As mentioned, in addition to the use of arithmetic strategies, another ingredient that has been hypothesised to be involved in the groupitizing effect, is “subitizing”. Subitizing is a phenomenon whereby estimations up to set sizes of about 4–5 items, are extremely fast and almost errorless^22–24^. Beyond this limit, performance rapidly deteriorates, as the number of elements increases. There is evidence suggesting that groupitizing leverages on subitizing. For example, both groupitizing and subitizing require attentional resources^16^ and a recent study has shown that it is possible to groupitize small groups of items, suggesting a link between the two phenomena^25^. Interestingly, subitizing has recently been shown to be unrelated to mathematical abilities of typically developing children^26^ and not impaired in a sample of individuals with dyscalculia^27^. The fact that groupitizing was not found in the present study to be impaired in individuals with dyscalculia is in line with the idea that it might be largely based on subitizing, which is also likely not impaired in dyscalculia and unrelated to math skills.

Some note of cautions should be made at this point. Despite similar groupitizing advantages has been found for both approximate estimation^17–20,25^ and exact serial counting tasks^14,16^, they can still rely on different mechanisms. It is therefore important to underline that, as we only measured groupitizing with an approximate estimation task, our results cannot be directly generalised to counting tasks. In the current study, *RTs* were measured between the stimulus offset and the instant when the experimenter pressed the response button as a consequence of participant’s vocalisation. Despite it is clear that following this approach we obtained RT estimates that also included the experimenter’s RTs, such procedure was considered the best choice to make the task as easy as possible for children. More, as we always had the very same experimenter to collect the data, the RTs estimates might, at worst, been affected by a constant bias playing little or no role in accounting for the reported effects.

Taken together, the current results and the existing literature, suggest that the link between groupitizing and mathematical abilities cannot be taken for granted, calling for further detailed investigations on the factors underlying this perceptual phenomenon across different developmental stages.

## Methods

### General procedures

The experimental procedures were approved by the local ethic committee (*Comitato Universitario di Bioetica Università degli Studi di Perugia, Prot. n. 908 del 12-1-2021*). The research was performed in accordance with the Declaration of Helsinki and informed consent was obtained from all parents prior to the experiment. Visual stimuli were created with Psychophysics toolbox for Matlab and displayed on a 60 Hz—13″ screen monitor (Microsoft Surface pro) placed at a viewing distance of 57 cm. Subjects were tested in a quiet, dimly light room. Numerosity perception and math abilities were usually tested on the same day.

### Participants

A total of 38 Italian children participated in this study: 19 diagnosed with developmental dyscalculia (DD) aged 11–19 years (mean 14 years, SD 2) and 19 typically developing children (TD, mean 13 years, SD 1.6) matched for age (t_(36)_ = 1.7, p = 0.09). DD met Diagnostic and Statistical Manual of Mental Disorders, Fifth Edition (DSM-V) criteria for dyscalculia (severe difficulties in math reasoning and calculation, severe school difficulties below those expected by the chronological age, early onset of math difficulties and math difficulties that cannot be explained by intellectual disabilities, sensory and/or neurological deficits as well as by psychosocial adversities, lack of language knowledge or inadequate education). Eleven DD also had a current diagnosis of dyslexia (one associated with ADHD, six associated with dysorthography). All the typically developing children had no medical or psychiatric diagnosis, as reported by parents and teachers.

### Neuropsychological measures

#### Reading

Reading decoding abilities were assessed by an Italian battery requiring a reading aloud word-list^28^. The scores were transformed into age standardized z-scores according to the normative data provided by the test manual.

#### Intelligence

The group with dyscalculia performed a full IQ scale (WISC-IV). Reasoning abilities in the control group were assessed with a non-verbal test (Progressive Raven Matrices). In the group with dyscalculia, non-verbal reasoning abilities were indexed by a sub score derived from the WISC-IV (perceptual reasoning index, IRP).

#### Mathematical abilities

Math abilities were assessed by a comprehensive Italian battery for the diagnosis of dyscalculia^29^. Both groups completed 9 sub-tests: (1) Counting. The child counts aloud between 80 and 140 in ascending order and the experimenter measures the time. The child is than asked to count in a descending order from 140 until he/she reaches the time taken in the ascending count. The score is the number of numerals stated correctly. (2) Numbers reading. The child reads aloud 48 Arabic numbers arranged in four different lists, each composed of 12 integer numbers of three, four, five, or six digits. The score is the total of numerals stated correctly within 60 s. (3) Numbers writing. The child writes in Arabic format 18 spoken number words (three to six digits) named by the experimenter. The score is the total of numeral written correctly. (4) Mental multiplications. The child is asked to solve 18 multiplication tables (e.g. 2 × 3) read by the examiner in a random order and allowing a maximum of 3 s to answer. The score is the total number of correct answers provided within 3 s. (5) Mental calculation. The child is asked to solve 9 addition and 9 subtractions (e.g. 27 + 14, 43 − 12) read by the examiner and allowing a maximum of 30 s to answer. The score is the total number of correct answers provided within 30 s. (6) Fast mental calculation. The child is provided with a sheet of 40 operations (addition, subtraction, division, multiplication e.g. 100 ÷ 25, 50 × 11, 15 ÷ 3, 24 − 6) and asked to perform as many as possible in a maximum of 2 min. Operations are not allowed to be solved by writing. The score is given by the number of correct operations performed within the maximum time. (7) Choose the largest. For 18 trials the child chooses the largest number among a set of three Arabic numbers (one to five digits). Both accuracy and speed are measured. (8) Insert numbers. For 18 trials the child positions a number (one to six digits) in one of four possible positions among three other numbers. Both accuracy and speed are measured. (9) Approximate mental calculation. The child is given a sheet with 18 operations (addition, subtraction, division, multiplication e.g. 28 ÷ 7, 215 + 55, 820 ÷ 20). For each operation, four answer options are provided. The child is asked to mark the right answer and perform as many operations as possible in 2 min. In this test the child is explicitly told to look for which of the results is the most plausible, forcing an approximation strategy. The score is given by the number of correct operations performed within the maximum time. The 9 scores were transformed into age standardized z-scores according to the normative data provided by the test manual. The z-scores were averaged to obtain an aggregate math index.

### Groupitizing task

Groupitizing was measured with a numerosity estimation task. Stimuli were arrays of squares (0.4° × 0.4°, white squares within black borders to balance luminance) displayed for 500 ms in each trial. Squares could not overlap and were constrained to fall within a 13° × 13° virtual square area. In the unstructured conditions, the position of each square was randomly selected from 154 possible positions (within the stimulus area), being the centers of equally spread sectors within the 13 × 13° area (each grid 1 × 1°). For the spatially grouped condition, stimuli were arranged in 4 possible groups of 12 possible positions. Each group (spanning over a max area of 4 × 2°) was located in one quadrant and centered at 5° from the central fixation point. Each group was first randomly assigned to one quadrant (between 1 and 4), then the individual items’ positions were randomly selected between one of the 12 in the selected quadrant. Within each quadrant, the maximum center-to-center distance between each element was 4° and the minimum was 1°.

Each trial started with a central fixation point that remained on screen for the entire experiment. After 500 ms a stimulus was displayed, followed by a blank screen. Participants estimated verbally the numerosity of the squares-array. The experimenter hit the spacebar when the participant responded (used to calculate response times), then entered the response on the numeric keypad, which initiated the following trial. There was no time pressure on responses. Response time was measured from the stimulus offset to the beginning of vocalization and were calculated for both right and wrong response trials. Each condition was tested in separate blocks, and participants were never explicitly informed about the grouping cue.

Numerosity levels ranged from 5 to 10 (grain of 1, resulting in 6 numerosity levels). Following previous studies^14,15,17,19,20^ in the structured conditions, each numerosity was organized into a few clusters (between 2 and 4), each containing a few items (between 1 and 4), resulting in the following configurations: 2-2-1; 3-3; 2-2-2; 3-3-1; 3-3-2; 2-2-2-2; 4-4; 4-4-1; 3-3-3; 3-3-3-1 (these cluster were those providing the most robust results in the mentioned previous studies). As numerosities 5 and 10 were not analyzed (see “Data analysis”), each grouped pattern comprised a minimum of 2 and a maximum of 4 clusters. All clusters contained between 1 and 4 elements. On each trial, a given numerosity and configuration pattern were randomly selected. Each participant completed about 80 trials for each of the two conditions roughly equal across numerosity levels (around 6300 trials in total across participants and conditions). To minimise the possibility of adding non-perceptual noise, grouped and unstructured stimuli were tested on separate blocks.

### Data analysis

Since participants were explicitly informed about the numerical range (5–10), we eliminated the two extreme numerosities from the analyses. We controlled for response outliers by eliminating trials with *RTs* and/or response time longer or shorter than 3 z-scores, calculated separately for each numerosity level and participant (93 trials in total).

For each participant, and separately for each numerosity, we calculated the average perceived numerosity, the responses standard deviation and the mean response time. The standard deviation divided by the physical numerosity yields the coefficient of variation (*CV*), a dimensionless index of precision that allows comparison and averaging of performance across numerosity

1 $$ CV = 1/n\mathop \sum \limits_{i = 1}^{n} \frac{{\sigma_{i} }}{{N_{i} }} $$

where *n* is the number of analyzed numerosities (= 4), indexed by *i. N*_*i*_ is the *i*th numerosity and *i* the standard deviation of responses to numerosity *i*.

Performance advantage induced by groupitizing was indexed by the between numerosity average difference between unstructured and grouped condition.

2 $$ Advantage = CVu\,\, or\,\, RTu - CVg\,\, or\,\, RTg $$

where *CV*_*u*_ and *RT*_*u*_ are the coefficient of variation and response time for the unstructured stimuli condition while *CV*_*G*_ and *RT*_*G*_ are the coefficient of variation and response time for the grouped conditions.

Data were analyzed by Repeated Measures ANOVAs, *t* test and Pearson correlations. Frequentist statistics were supplemented with Bayesian statistics, calculating Bayes Factors, the ratio of the likelihood of the alternative to the null hypothesis, and reporting them as base ten logarithms (Log10 Bayes Factors, *LBF*). For RM-ANOVA with report LBF_inclusion_ indicating how much the data are likely to occur from a model including that specific factor (or interaction), compared to models not including them. By convention, *LBF* > 0.5 is considered substantial evidence in favour of the alternative hypothesis (difference between groups in this case) and *LBF* < − 0.5 substantial evidence for the null hypothesis (no difference). Absolute values greater than 1 are considered strong evidence, and greater than 2 definitive. Data were analyzed by JASP (Version 0.8.6) and Matlab (R2017b) software.
